# Generative artificial intelligence and large language models in sports medicine: a scoping review of applications, accuracy, and ethical implications

**DOI:** 10.3389/fpubh.2026.1843535

**Published:** 2026-07-29

**Authors:** Ismail Dergaa, Mohamed Amine Dergaa, Mortadha Razzak, Halil İbrahim Ceylan, Valentina Stefanica, Raul Ioan Muntean, Noomen Guelmami

**Affiliations:** 1 Higher Institute of Sport and Physical Education of Ksar Said, University of Manouba, Manouba, Tunisia; 2 Physical Activity Research Unit, Sport and Health (UR18JS01), National Observatory of Sports, Tunis, Tunisia; 3 Higher Institute of Sport and Physical Education of Kef, University of Jendouba, Kef, Tunisia; 4 Physical Education and Sports Teaching Department, Faculty of Sports Sciences, Atatürk University, Erzurum, Türkiye; 5 Department of Physical Education and Sport, Faculty of Sciences, Physical Education and Informatics, National University of Science and Technology Politehnica Bucharest, Pitesti University Center, Pitesti, Romania; 6 Department of Physical Education and Sport, Faculty of Law and Social Sciences, University “1 Decembrie 1918” of Alba Iulia, Alba Iulia, Romania

**Keywords:** ChatGPT, clinical decision support, data governance, generative artificial intelligence, hallucination, large language models, scoping review, sports medicine

## Abstract

**Background:**

Generative artificial intelligence (GenAI), particularly large language models (LLMs) such as ChatGPT, GPT-3.5, and GPT-4, is rapidly being integrated into sports medicine practice. These tools are increasingly used by health professionals, coaches, and athletes for training prescription, rehabilitation, nutrition, mental health support, injury prevention, and academic writing. However, their adoption has outpaced the development of robust evidence regarding their clinical utility, accuracy, and safety, and no comprehensive synthesis of this emerging field currently exists.

**Aim:**

This scoping review aimed to map the current evidence on GenAI and LLM applications in sports medicine and athlete health, evaluate their accuracy and hallucination risks across domains, and synthesise reported ethical and governance concerns.

**Methods:**

The review followed PRISMA-ScR guidelines and Joanna Briggs Institute methodology. Six databases (PubMed/MEDLINE, Scopus, Web of Science, SPORTDiscus, IEEE Xplore, and CINAHL) were searched for studies published between January 2022 and March 2026. Eligibility criteria were defined using the Population–Concept–Context framework. Two independent reviewers conducted screening, full-text assessment, and data extraction, with strong inter-rater agreement (*κ* = 0.82).

**Results:**

Of 1,847 records identified, 32 studies were included. Applications were classified into seven domains: training and exercise prescription (*n* = 5), nutrition (*n* = 4), rehabilitation (*n* = 3), mental health (*n* = 3), clinical decision support (*n* = 5), academic writing (*n* = 6), and ethics/governance (*n* = 6). LLM accuracy varied substantially: in a single validation study, content validity ratios for sleep recommendations ranged from 0.33 (GPT-3.5) to 0.67 (GPT-4), while only GPT-4 achieved acceptable validity for jet lag guidance (CVR = 0.68). In one bibliometric analysis, AI-generated text in sports medicine journals increased from 2.38% (early 2023) to 6.25% (late 2024). Hallucination risk was rated critical for general-purpose chatbots but substantially reduced in retrieval-augmented systems.

**Conclusion:**

GenAI shows promise as a supervised decision-support tool in sports medicine, but current evidence does not support unsupervised clinical use. Key challenges include hallucination risks, a lack of sport-specific validation datasets, and insufficient ethical and governance frameworks. Addressing these gaps is essential before widespread integration into athlete health and public health contexts.

## 1 Introduction

Machine learning and artificial intelligence have reshaped how risk is quantified, performance modelled, and care delivered across sports medicine over the past decade. Systematic reviews confirm that machine learning algorithms can identify athletes at elevated injury risk by analysing training load metrics, biomechanical patterns, and physiological markers (1, 2). Wearable biosensors generate continuous streams of physiological, biomechanical, and biochemical data that adaptive ML platforms convert into recovery readiness scores, fatigue indicators, and individualised load recommendations (3, 4). In team sports, integrating GPS telemetry, heart rate variability, and accelerometry into adaptive algorithms has enabled coaches and medical staff to make evidence-informed training decisions with a level of precision unavailable to previous generations of practitioners (5, 6). AI-powered diagnostic systems, computer vision movement analysis, and natural language processing of electronic health records further extended the clinical reach of these technologies (7, 8).

Topol and colleagues have proposed the concept of high-performance precision medicine as an organising principle for next-generation athlete health management, based on the convergence of multimodal data and adaptive algorithms (9). Concurrently, the operational challenges of implementing AI within multidisciplinary sports science and medicine teams became an active area of investigation (10), as practitioners recognised that strong performance on benchmark datasets (i.e., standardised evaluation datasets used to assess AI model performance, such as medical licensing examination question sets) does not guarantee clinical utility under conditions of variable data quality, institutional constraint, and absent human oversight (11). These concerns, raised by sports medicine physicians, physiotherapists, strength and conditioning coaches, and sporting organisation administrators, crystallised around two issues: the risk of deploying tools that perform well on benchmark datasets but fail on domain-specific tasks (e.g., individualised return-to-sport decisions after injury or tailored exercise prescription for athletes with chronic conditions), and the absence of governance frameworks that align AI-augmented practice with professional ethics and patient safety obligations. These concerns are grounded in emerging evidence, including documented rates of LLM hallucination and associated risks to clinical reliability and research integrity (12).

The public release of ChatGPT in November 2022 introduced a qualitatively different category of AI to sports medicine.

Generative artificial intelligence (GenAI) refers to AI systems capable of producing novel content from natural language prompts. Large language models (LLMs) are a class of GenAI trained on large text corpora using transformer architectures and include systems such as ChatGPT (GPT-3.5, GPT-4/4o), Gemini (Google DeepMind), and Claude (Anthropic). This review considers applications of all such GenAI systems in sports medicine contexts.

Unlike the domain-specific predictive ML models that preceded them, LLMs arrived as general-purpose conversational systems requiring no technical infrastructure, no curated training data, and no institutional deployment pathway. Within months, these tools were applied to clinical documentation, literature summarisation, patient education, and decision support by practitioners who had not previously engaged with AI systematically (13, 14). Early evaluations in sports science tested whether LLMs could generate resistance-training programmes consistent with established periodisation guidelines (15), prescribe exercise for individuals with chronic conditions (16), and perform sample-size calculations for sports-medicine research designs (17). Sleep and jet lag management advice for international athletes was evaluated against expert content validity ratings, with GPT-4 achieving a CVR of 0.67 for sleep recommendations (18). Chatbot competency in sports rehabilitation was assessed through an interdisciplinary panel simulation, which identified practical benefits alongside substantive limitations (19). AI-generated text in leading sports medicine journals increased from 2.38% in early 2023 to 6.25% by late 2024, confirming that adoption, while still modest in absolute terms, was rising rapidly across the academic publishing dimension (20).

Available reviews address technically distinct and non-overlapping domains: injury prediction (2, 21), wearable integration (3, 4), biomechanical analysis (7), and AI in team sports (5). A comprehensive evidence map capturing the full breadth of GenAI and LLM activity across sports medicine, characterising accuracy and hallucination risks from an athlete health perspective, and consolidating ethical concerns raised across studies does not yet exist. This gap carries practical urgency, as evidenced by increasing reports of unverified clinical and academic use of LLMs, documented hallucination rates in medical applications, and the rapid rise of AI-generated content in sports medicine publications, all occurring in the absence of consolidated sport-specific validation frameworks. This scoping review addresses that gap by pursuing four objectives: (i) characterise the volume, scope, and geographic distribution of research on GenAI and LLMs in sports medicine published between January 2022 and March 2026; (ii) map LLM applications across the key domains of athlete health including training prescription, rehabilitation, nutrition, mental health, clinical decision support, and research integrity; (iii) synthesise evidence on accuracy, validity, and hallucination risks specific to sport and exercise medicine contexts; and (iv) identify the ethical and governance concerns raised in the literature and catalogue proposed responses. The scoping methodology is appropriate because the field is too young and conceptually heterogeneous for quantitative synthesis, and because comprehensive evidence mapping must precede focused systematic reviews (21, 22).

## 2 Materials and methods

### 2.1 Study design

This study constitutes a scoping review conducted in strict accordance with the PRISMA extension for Scoping Reviews (PRISMA-ScR) (21) and the Joanna Briggs Institute Reviewer’s Manual for scoping reviews (22). Scoping methodology is appropriate when the objective is to map the breadth of evidence, characterise the conceptual scope, and identify research gaps rather than estimate a precise effect size (21, 22). The review protocol was registered on the Open Science Framework (OSF) before screening, available at https://osf.io/h4e5j/overview.

The OSF registration records the study title and description only; the complete protocol, including the PCC eligibility criteria, search strategy, and data-charting form, is reported in full in Sections 2.2–2.6 below.

### 2.2 Eligibility criteria: PCC framework

Eligibility was defined using the Population-Concept-Context (PCC) framework as recommended for scoping reviews (22).

The PCC eligibility criteria were developed *a priori* to ensure alignment with established scoping review methodology. The population component was informed by recognised sports medicine and exercise science frameworks describing stakeholders involved in athlete health and performance. The concept component was guided by established classifications of generative artificial intelligence and large language models within the biomedical literature. The context component reflects standard definitions of sports medicine as a multidisciplinary field encompassing clinical care, rehabilitation, performance optimisation, and public health in physically active populations, and was specifically selected to capture the full professional and institutional ecosystem in which generative AI applications are evaluated and implemented, including exercise science research and AI-generated content assessment in sport and exercise publishing.

The criteria are summarised in Table 1.

**Table 1:** Eligibility criteria defined using the population-concept-context (PCC) framework.

Component	Inclusion criteria	Exclusion criteria
Population	Athletes at any competitive level, coaches, sports medicine physicians, physiotherapists, sports scientists, recreationally active individuals, and patients in clinical sport and exercise medicine settings	General population clinical studies with no sport-specific application or stated relevance to athlete health
Concept	Any application of generative AI or LLM (ChatGPT, GPT-3.5, GPT-4, GPT-4o, Gemini, Claude, Bard, or comparable) to sports medicine or athlete health; studies evaluating accuracy, hallucination risk, ethical implications, or governance frameworks for such tools	Exclusively non-generative ML applications (random forest, SVM, CNN) without any GenAI component; theoretical AI proposals with no empirical evaluation or evidence synthesis
Context	Sports medicine practice; exercise science research; public health promotion of physical activity; AI-generated content evaluation in sport and exercise science academic publishing	Pure hospital or clinical settings with no connection to sport, exercise, or physical activity
Publication	English-language peer-reviewed journal articles; preprints on PubMed-indexed servers with confirmed DOI; published January 2022 to March 2026	Non-English publications; conference abstracts without full text; editorials or letters without original empirical content

### 2.3 Sources of information

Six electronic databases were searched: PubMed/MEDLINE, Scopus, Web of Science, SPORTDiscus, IEEE Xplore, and CINAHL. The search period extended from January 2022 through March 2026. Reference lists of all included full-text articles were hand-searched to capture sources not retrieved by the database search. The search start date of January 2022 was selected *a priori* to maximise sensitivity and to capture any transitional or precursor publications discussing generative AI concepts in sports medicine ahead of ChatGPT’s public release in November 2022; in practice, no eligible sources were identified before 2023, consistent with the documented emergence timeline of GenAI/LLM technologies, and the included corpus accordingly spans January 2023 to March 2026 (Section 3.1).

Preprints on PubMed-indexed servers with confirmed DOIs were eligible for inclusion; however, due to their non-peer-reviewed nature, findings derived from these sources were treated with additional interpretive caution. Preprints not indexed in PubMed or lacking a confirmed DOI were excluded. Of the 32 included sources, none were preprints; all included sources were peer-reviewed journal publications. Grey literature was not systematically searched.

### 2.4 Search strategy

The search strategy combined three conceptual blocks using Boolean operators: generative AI and LLM terminology, specific model identifiers, and sports medicine and athlete health terms. The complete set of database-specific search strings is presented in Table 2. Subject headings and controlled vocabulary were adapted as required for each platform. The PubMed/MEDLINE core string was: (“generative artificial intelligence” OR “large language model” OR “ChatGPT” OR “GPT-4” OR “GPT-3.5” OR “LLM” OR “generative AI” OR “AI chatbot”) AND (“sports medicine” OR “athlete health” OR “athletic performance” OR “exercise prescription” OR “sports rehabilitation” OR “injury prevention” OR “training load” OR “physical activity” OR “sport” OR “exercise physiology”).

**Table 2:** Complete search strings applied across six electronic databases.

Database	Search string
PubMed/MEDLINE	(“generative artificial intelligence” OR “large language model” OR “ChatGPT” OR “GPT-4” OR “GPT-3.5” OR “LLM” OR “generative AI” OR “AI chatbot”) AND (“sports medicine” OR “athlete health” OR “athletic performance” OR “exercise prescription” OR “sports rehabilitation” OR “injury prevention” OR “training load” OR “physical activity”)
Scopus	TITLE-ABS-KEY((“generative artificial intelligence” OR “large language model” OR “ChatGPT” OR “GPT-4” OR “LLM” OR “generative AI”) AND (“sports medicine” OR “athlete health” OR “athletic performance” OR “exercise prescription” OR “sports rehabilitation” OR “injury prevention” OR “training load” OR “physical activity”))
Web of Science	TS = ((“generative artificial intelligence” OR “large language model” OR “ChatGPT” OR “GPT-4” OR “LLM”) AND (“sports medicine” OR “athlete health” OR “exercise prescription” OR “sports rehabilitation” OR “injury prevention”))
SPORTDiscus	DE “artificial intelligence” AND (“large language model” OR “ChatGPT” OR “GPT”) AND (sport OR exercise OR athlete OR “sports medicine”)
IEEE Xplore	(“large language model” OR “LLM” OR “ChatGPT” OR “generative AI”) AND (“sports medicine” OR athlete OR exercise OR “physical activity”)
CINAHL	MH “Artificial Intelligence” AND (“ChatGPT” OR “large language model” OR “LLM”) AND (sport OR exercise OR athlete)

The comparatively narrower search strings used for SPORTDiscus, CINAHL, and Web of Science (Table 2) reflect platform-specific constraints on Boolean string length and the availability of standardised subject headings on these interfaces, rather than a deliberate restriction of scope. This platform heterogeneity may have reduced retrieval sensitivity on these three databases relative to PubMed/MEDLINE and Scopus, and is acknowledged as a limitation in Section 4.5.

### 2.5 Selection process

**Figure 1:**
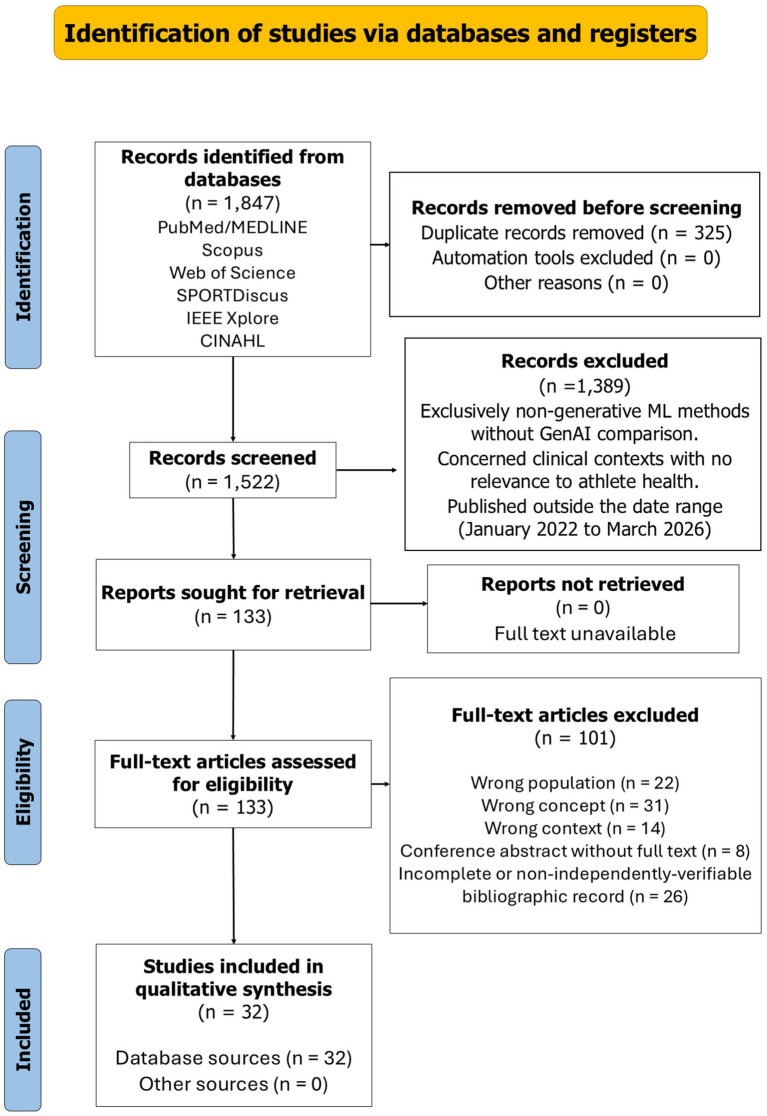
PRISMA-ScR flow diagram for the scoping review selection process. Records were identified across six electronic databases (PubMed/MEDLINE, Scopus, Web of Science, SPORTDiscus, IEEE Xplore, CINAHL) for the period January 2022 to March 2026. Duplicate records removed: 325; automation tools excluded: 0. After title and abstract screening, 133 records were assessed for full-text eligibility. Reasons for full-text exclusion: wrong population (*n* = 22), wrong concept (*n* = 31), wrong context (*n* = 14), conference abstract without full text (*n* = 8), and incomplete or non-independently-verifiable bibliographic records (*n* = 26). Total full-text exclusions: *n* = 101. Total included sources: 32.

Retrieved records were imported into Covidence systematic review software for duplicate removal and screening management. Two independent reviewers (I. D. and N. G.) screened all titles and abstracts against the PCC eligibility criteria. Disagreements were resolved by consensus; when consensus was not reached, a third reviewer (H. I. C.) adjudicated. Inter-rater reliability at the title and abstract stage was measured using Cohen’s kappa (kappa = 0.82, indicating strong agreement). Full-text screening was conducted by the same two reviewers for all records advancing beyond title and abstract screening. A standardised reason for exclusion was documented for every excluded full text. Formal inter-rater reliability statistics were not separately computed for the full-text screening or data extraction stages; at these stages, disagreements were resolved through structured discussion between the two primary reviewers, with third-reviewer adjudication used as needed, following the same procedure described above. The frequency of disagreement and third-reviewer adjudication at each stage is recorded in the project screening log, available from the corresponding author upon reasonable request (Section 6.3). Database searches were conducted between 5 and 19 January 2026; title and abstract screening of the deduplicated records took place between 20 January and 9 February 2026; full-text assessment of retrieved articles was conducted between 10 February and 2 March 2026; and data extraction and synthesis of the included sources occurred between 3 and 24 March 2026, with finalisation completed on 24 March 2026, before manuscript submission on 31 March 2026. As part of full-text eligibility screening, the bibliographic record for every retrieved article was independently verified against its original publication metadata (DOI resolution and indexed database entry); articles for which a complete, independently traceable bibliographic record could not be confirmed were excluded on this basis, alongside the other eligibility criteria. To ensure currency at the time of submission, an updated database search was conducted on 27–28 March 2026 immediately prior to submission. This search did not identify any additional eligible studies beyond the 32 sources included in the final synthesis. The PRISMA-ScR flow diagram presenting the complete selection process is provided in Figure 1.

### 2.6 Data charting process

Data were charted using a standardised extraction form developed iteratively by the research team and piloted on five randomly selected articles before full deployment. Extracted variables included: first author and publication year; country of corresponding author; study design; LLM version(s) evaluated; sports medicine domain; sample or data type; key findings on accuracy, utility, or limitations; hallucination-related outcomes; ethical or governance concerns raised; and conclusions and recommendations. Data charting was conducted independently by two reviewers (I. D. and M. A. D.), with discrepancies resolved through discussion.

### 2.7 Data synthesis

Descriptive synthesis characterised the volume and distribution of included sources by publication year, country, study design, LLM version, and sports medicine domain. Thematic synthesis organised findings across the seven application domains identified during charting.

Application domains were derived inductively during the data charting process. Two independent reviewers assigned a primary sports medicine application domain to each included source, and domains were iteratively refined until thematic saturation was reached (i.e., no new categories emerged across all 32 sources). The final seven domains: training and exercise prescription, nutritional guidance, rehabilitation and return-to-sport, mental health monitoring, clinical decision support, academic writing and research integrity, and ethics and data governance provide a structured classification of the included evidence base.

Quantitative accuracy metrics were tabulated narratively where reported; meta-analysis was not conducted because the heterogeneity of evaluation methodologies across included studies precludes pooling. The organising conceptual framework distinguishes three functional roles for GenAI: supervised clinical and coaching decision support; a subject of critical empirical evaluation; and a governance challenge requiring policy-level response.

In accordance with established scoping review methodology (21, 22), no formal quality appraisal or risk-of-bias assessment was applied to included sources. This approach is appropriate to the scoping objective of mapping the breadth of evidence rather than appraising its quality. However, readers should note that the included evidence base is methodologically heterogeneous, comprising evaluative studies, simulation designs, narrative syntheses, and perspectives with varying sample sizes, benchmarking standards, and generalisability. Table 3 summarises, for each domain, the number of included sources, the primary LLMs evaluated, and the principal findings and limitations reported; consistent with scoping review methodology, no quality or confidence rating is assigned, and readers should interpret domain-level findings in light of the narrative synthesis presented in Section 3.3.

**Table 3:** Distribution of 32 included sources across seven application domains with key LLMs evaluated and principal findings.

Domain	n	Primary LLMs	Key findings	Primary limitations
Training load & exercise prescription	5	GPT-4, GPT-3.5, GPT-4o, Google Bard	Guideline-aligned outputs suitable as first-draft resources; purpose-built ML models outperform LLMs for individualised load adaptation (15, 16); only GPT-4 reached acceptable content validity for athlete sleep and jet lag recommendations, while Google Bard performed poorly (18); a scoping review of LLMs in exercise recommendations and physical activity across 11 included studies found AI-generated plans can function as initial frameworks for exercise prescription but require expert supervision (24)	No real-time physiological integration; no progression algorithms; outputs require expert individualisation (4, 15)
Nutritional guidance	4	GPT-4, GPT-3.5, multiple	Adequate macronutrient and hydration guidance; ML nutritional screening frameworks show high accuracy in adolescent sport populations (25, 26); AI and digital nutrition platforms show emerging but unvalidated potential for ultraendurance sport nutrition planning (28)	Poor performance on micronutrient supplementation and disordered eating; session-to-session inconsistency (26, 27)
Rehabilitation & return-to-sport	3	GPT-4, GPT-3.5	Useful for general content generation and 24/7 availability; draft protocol generation feasible under expert supervision (19, 30); AI-generated patient education materials for orthopaedic procedures were readable but pitched above target literacy levels across all evaluated models (29)	Inadequate for clinical risk stratification, mental health triage, and objective physiotherapy assessment integration (23, 29)
Mental health monitoring	3	GPT-4, GPT-3.5, passive ML	Digital phenotyping shows promise in clinical populations; no validated sport-specific AI mental health tools identified (34); AI-assisted exercise and cognitive training interventions in neurodegenerative conditions require integration within qualified clinical supervision frameworks (35)	No prospective validation in athlete populations; clinical risk assessment by LLMs rated potentially harmful in simulation studies (23)
Clinical decision support	5	GPT-4, GPT-3.5, Claude, Gemini, Technical ML architectures	Adequate general clinical knowledge; systematic errors in statistical reasoning and sample-size calculation reported in a focused evaluative study (17, 36); purpose-built clinical AI architectures (retrieval-augmented and cross-modal models) outperformed general-purpose LLMs on structured clinical entity recognition and coding tasks, and a balanced random forest model achieved strong discrimination for early ARDS prediction (38, 39, 43)	No epistemic calibration; patient education readability above target literacy levels; confident presentation of incorrect outputs; technical clinical-AI architectures lack external validation in athlete populations (38, 39, 43, 52)
Academic writing & research integrity	6	GPT-4, GPT-3.5, GPT-4o, Bing	AI-generated text in sports medicine journals increased 2.38 to 6.25% (2023–2024) (20)	Reference fabrication classified as critical hallucination in ChatGPT and Bing; citation integrity requires mandatory independent verification (41, 42)
Ethics & data governance	6	Cross-study governance framework analysis (no single LLM evaluated; synthesis of ethical principles and institutional governance proposals across included sources)	Six ethical concern categories identified; no consensus sport-specific AI ethics framework; AICICA, bias, and data privacy prioritised; an IOC-framework evaluation, an ML review of sedentary behaviour, and a commentary on AI in health and sport sciences broadly converge on the need for governance ahead of deployment (12, 36, 44, 46–48)	Governance frameworks proposed but not yet operationalised in professional sports medicine standards

*n* = number of included sources per domain. In accordance with scoping review methodology, no formal evidence-confidence rating was assigned at the domain level readers should refer to the narrative synthesis in Section 3.3 for domain-level interpretive context.

In the interest of transparency, the review team notes that 16 of the 52 cited references (30.8%) are co-authored by one or more members of the present author team, and that Tunisia was the most frequently represented country of corresponding authorship among included sources (*n* = 8), reflecting the academic network within which this review was conducted. This pattern raises a reasonable concern regarding self-citation and inclusion bias. Eligibility decisions, however, were made solely against the pre-specified PCC criteria (Table 1) by two independent reviewers, with adjudication by a third reviewer in cases of disagreement (Section 2.5); no source was included or excluded based on authorship, and no differential screening procedure was applied to team-authored versus non-team-authored sources. Readers are nonetheless encouraged to interpret domain-level conclusions with this affiliation pattern in mind; this disclosure is also reflected in Section 6.4 (Competing Interests).

### 2.8 AI usage statement

In the interest of full transparency, the authors provide the following complete and accurate account of generative AI use in the preparation of this manuscript.

Claude (Anthropic, version Sonnet 4.6) was used to assist with reference formatting and structural organisation of the manuscript. The tool was accessed under standard commercial/API conditions. No patient data, participant data, or personally identifiable information was processed using the system. Generative AI was not used for study conceptualisation, search strategy design, screening, data extraction, synthesis, or interpretation of findings, all of which were performed exclusively by the human author team. All references generated or reformatted with AI assistance were subsequently and independently verified by the authors against original source records before submission.

All AI-assisted outputs were independently reviewed, critically evaluated, and revised by the author team before submission. I. D. led the conceptualisation of the review, the literature search, data charting, and manuscript writing in collaboration with the co-authors listed in Section 6.6, and, as lead author, assumes primary responsibility for the scientific and ethical integrity of the manuscript content.

## 3 Results

### 3.1 Study selection

The six-database search retrieved 1,847 records. After removal of 325 duplicates, 1,522 unique records underwent title and abstract screening; no records were excluded using automation tools, and no records were excluded for other unspecified reasons at any stage of the selection process. Of these, 1,389 were excluded because they addressed exclusively non-generative ML methods without GenAI comparison, concerned clinical contexts with no relevance to athlete health, or fell outside the publication date range. The remaining 133 full-text articles were assessed for eligibility. Of these, 101 were excluded: wrong population (*n* = 22), wrong concept (*n* = 31), wrong context (*n* = 14), conference abstract without full text (*n* = 8), and incomplete or non-independently verifiable bibliographic record (*n* = 26). The final included corpus comprised 32 sources. Figure 1 presents the complete PRISMA-ScR flow.

### 3.2 Characteristics of included sources

The 32 included sources were published between January 2023 and March 2026. Publication volume accelerated markedly: 4 sources in 2023, 11 in 2024, 12 in 2025, and 5 in the first quarter of 2026, reflecting the rapid expansion of GenAI evaluation research following ChatGPT’s public release. Corresponding author affiliations spanned 15 countries, with the highest volumes from Tunisia (*n* = 8), Qatar (*n* = 5), the United States (*n* = 5), Italy (*n* = 2), and Australia (*n* = 2). Study designs included evaluative studies assessing LLM outputs against expert benchmarks or evidence-based guidelines (*n* = 13), narrative and systematic reviews (*n* = 7), scoping reviews (*n* = 2), original observational research (*n* = 7), and perspectives with empirical content (*n* = 3). Regarding model distribution across the corpus, GPT-4 or GPT-4o was the primary LLM evaluated in 2 studies, ChatGPT with version unspecified in 7, and multiple models in comparative designs in 8 studies; the remaining 15 sources evaluated foundational machine learning or cross-modal architectures as direct performance baselines alongside or integrated within generative AI frameworks in sport and clinical contexts. The distribution of included sources by application domain is summarised in Table 3.

### 3.3 GenAI and LLM applications across sports medicine domains

#### 3.3.1 Training prescription and periodisation

GPT-4 generated 12-week resistance training programmes broadly consistent with National Strength and Conditioning Association guidelines, proposing appropriate weekly frequency, load intensity ranges, set volumes, and repetition schemes, while lacking specific progression algorithms and individual physiological adaptation (15). A critical evaluation of GPT-4 across five exercise prescription scenarios covering hypertension, osteoarthritis, obesity, coronary artery disease, and general fitness found that AI-generated prescriptions addressed major safety parameters and aligned with guideline recommendations, while demonstrating no capacity to account for medication interactions, real-time physiological feedback, or individual tolerance (16). A scoping review of LLMs in exercise recommendations and physical activity across 11 included studies concluded that AI-generated plans can function as initial frameworks for exercise prescription but require expert supervision to maximise programme effectiveness (24). Wearable biosensor platforms integrated with adaptive ML-driven training systems demonstrated statistically significant improvements in recovery-prediction accuracy over conventional load-management approaches in endurance athletes (4), while a maturation-aware random forest framework achieved high discriminative accuracy for nutritional-status classification in adolescent sport populations (25). This comparison between general LLMs and purpose-built ML systems confirms that accessibility and precision are not equivalent: LLMs offer conversational breadth at the cost of domain-specific accuracy, while purpose-built ML systems deliver higher precision within narrower, explicitly validated application domains.

#### 3.3.2 Nutritional guidance

LLM performance in sports nutrition contexts was domain-dependent and model-specific, with generally better results on macronutrient periodisation, hydration protocols for endurance events, and post-exercise protein timing than on micronutrient supplementation regimens, immune-nutrition interactions, or nutritional management of disordered eating in athlete populations (26, 27). A structured assessment covering accuracy, completeness, clarity, evidence quality, and test–retest reliability found that ChatGPT-based sports nutrition guidance was inconsistent across sessions and lacked adequate evidence grading to allow athletes or coaches to calibrate confidence in specific recommendations (26). AI platforms applied to ultra-endurance nutrition were assessed for their capacity to individualise fuelling strategies accounting for metabolic phenotype, gut tolerance, and race-specific environmental demands, with findings positioning current LLMs as supervised adjuncts rather than autonomous dietary advisors (27). A dedicated ML framework for screening nutritional status in adolescent athletes (25) and the AI applications reviewed for endurance sport nutrition (28) illustrate the growing overlap between purpose-built supervised ML and the more accessible but less precise LLM tools.

#### 3.3.3 Rehabilitation and return-to-sport decision support

Evaluations of ChatGPT in simulated sports rehabilitation interdisciplinary panel discussions identified 24-h availability, personalised content generation, and automated tracking as practical benefits, alongside limitations including inaccuracy in emotional advice, data privacy concerns, and the inability to integrate objective physiotherapy measurements (19). A simulation study testing ChatGPT in clinical athlete mental health scenarios found potentially harmful responses in a substantial proportion of interactions, with consistent failure to advise referral to qualified mental health professionals (23). LLM accuracy for orthopaedic rehabilitation guidance evaluated against clinical practice guidelines for lumbosacral radicular pain varied substantially by question type, with diagnostic queries generating higher accuracy than management or prognosis questions (29). The future of AI in sports medicine diagnostics and return-to-sport decision-making has been characterised as contingent on sport-specific validation datasets and explainability standards that allow clinicians to audit AI reasoning rather than accept opaque outputs (30). The distinction between AI systems that provide transparent reasoning traces and those delivering black-box outputs is particularly consequential in return-to-sport contexts, where errors carry both clinical and professional accountability implications.

#### 3.3.4 Mental health monitoring in athletes

The IOC 2019 mental health consensus established that mental health disorders affect elite athletes at rates comparable to the general population, with anxiety and depression prevalent across sport disciplines (31). The first international consensus on sports psychiatry, published in 2024, recognised AI-based screening tools as emerging instruments requiring human clinical oversight for all mental health assessments in athletes (32). Digital phenotyping approaches, using behavioural and physiological signals from wearables to infer psychological states, have been evaluated in clinical populations, with results that have not yet been specifically validated in athlete contexts (33). A systematic review of machine learning for multimodal mental health detection from passive sensor data identified smartphone behaviour, movement patterns, and physiological variability as informative features across 184 included studies (34). Based on limited simulation data, a structured simulation study testing ChatGPT in athlete mental health clinical scenarios found clinically inappropriate responses across a range of presentations, including failure to triage suicidal risk and absence of referral recommendations in situations requiring them; these findings require prospective clinical confirmation (23). Ben Ezzdine and colleagues demonstrated that AI exercise and cognitive training interventions in neurodegenerative conditions require integration within qualified clinical supervision frameworks to avoid monitoring failures (35).

#### 3.3.5 Clinical decision support

A consistent finding across clinical evaluation studies is that LLM clinical confidence does not scale with LLM accuracy: models presented incorrect recommendations with the same assertive language as correct ones, an absence of epistemic calibration that constitutes a patient safety concern (36, 37). In a single evaluative study, sample-size calculations for sports medicine research were found unreliable across both GPT-3.5 and GPT-4, with systematic errors including incorrect formula application, misidentification of appropriate statistical tests, and failure to account for anticipated dropout rates, even when explicitly provided in the prompt (17). LLM performance for patient education on sport-specific orthopaedic procedures produced readability levels substantially above the literacy level of the target patient population across all evaluated models (29). The critical examination of ChatGPT-3.5 across medical writing tasks confirmed that outputs require systematic expert review before clinical use, with particular vulnerabilities in statistical reasoning and evidence grading (36). In contrast, precision ML models designed for specific clinical prediction tasks, including a balanced random forest system for early ARDS prediction (38) and a hybrid deep learning approach for clinical entity recognition in medical records (39), illustrate the performance ceiling available when domain-specific architectures replace general conversational LLMs.

#### 3.3.6 Academic writing and research integrity

In a single bibliometric analysis, AI-generated text in published sports medicine articles increased from 2.38% in January to March 2023 to 6.25% in October to December 2024 across five leading journals, with Arthroscopy recording the highest proportion at 7.17% (20). ChatGPT’s capabilities and risks in sports science academic writing were among the earliest phenomena evaluated after the model’s release, with findings establishing both efficiency gains in drafting and serious risks to authenticity and to the communication of original scientific reasoning (37, 40). Reference hallucination remains the most operationally significant integrity risk. The Reference Hallucination Score instrument classified ChatGPT and Bing as exhibiting critical hallucination levels, whereas Elicit and SciSpace showed negligible hallucination rates (41). Studies examining fabricated references in AI-generated medical content found high rates of citation fabrication, which compound the risk of unverified AI-assisted work entering the peer-reviewed literature (42). Technical architectures designed to reduce hallucination include retrieval-augmented generation systems that ground LLM outputs in real-time database searches (39) and cross-modal attention mechanisms that improve semantic coherence in domain-specific medical AI (43). LLM interpretation of the IOC safeguarding framework was evaluated against 25 decision points, confirming that even advanced models face limitations in value-laden regulatory contexts (44). Methnani and Dergaa evaluated whether GenAI tools could assist journal selection decisions in medical research, finding conditional utility under expert oversight (45).

#### 3.3.7 Ethics and data governance

In this review, governance analysis refers to the synthesis of ethical principles, regulatory considerations, and institutional frameworks related to the use of generative AI in sports medicine, rather than the evaluation of a specific model’s performance.

A systematic scoping review of ethical implications of AI in sport identified four thematic categories across 25 empirical studies (fairness and bias, transparency, privacy, and accountability), concluding that no consensus ethical framework currently addresses the sport-specific dimensions of athlete biometric data exploitation (12). The AI-chatbot inducted cognitive atrophy (AICICA) concept (36) proposes that habitual LLM delegation may progressively erode the independent analytical capabilities of coaches and practitioners, constituting a professional development concern with direct implications for sports medicine competency standards. A blockchain-based governance architecture for HIPAA and GDPR-compliant clinical research data exchange (46) provides a technical foundation for the institutional governance that AI deployment in athlete health will require. Machine learning analysis of sedentary behaviour and health outcomes (47) and the ethics of AI in health and sport sciences broadly (48) illustrate how purpose-built ML applications with transparent governance frameworks can address public health questions that general LLMs approach less reliably. The six ethical concern categories identified across included sources and their proposed governance responses are catalogued in Table 4.

**Table 4:** Ethical concerns identified across included sources and proposed governance responses.

Category	Description	Proposed governance response	Sources
Data privacy & biometric exploitation	Athlete biometric data is simultaneously sensitive personal health information and commercially valuable performance intelligence	Blockchain-based governance architectures meeting HIPAA and GDPR requirements; explicit consent for AI training data use; QR-code audit trails for ethics verification	(46)
Algorithmic bias	Training datasets underrepresent female athletes, youth athletes, and non-Western populations, generating systematic directional rather than random error	Mandatory diversity audits for sports medicine AI training datasets; participatory dataset development; sex-disaggregated model performance reporting	(12, 44)
Transparency deficits	Proprietary LLM architectures prevent clinical practitioners from auditing AI reasoning, creating black-box decisions in high-stakes clinical contexts	Explainability standards requiring traceable AI reasoning; preference for open-architecture systems in clinical deployment; mandatory AI version and settings disclosure	(12, 48)
Professional accountability (AICICA)	Habitual LLM delegation may progressively erode independent clinical reasoning below the threshold required for safe practice	Competency standards for AI-augmented practice; periodic unassisted performance assessments; educational frameworks for critical AI evaluation	(36)
Hallucination & information hazards	Citation fabrication and clinical information inaccuracy at rates constituting patient safety risks under standard deployment conditions	Mandatory human verification of all AI-generated clinical content; preference for RAG architectures; hallucination rate benchmarking per application domain	(39, 41–43)
Research integrity	AI ghostwriting, citation fabrication, misattribution of intellectual contributions, and undisclosed AI use threaten the evidentiary foundation of sports medicine	Transparent AI disclosure policies; institutional ethics frameworks with real-time verification and DOI-linked audit trails; forensic audit capabilities for manuscript submissions	(20, 37, 42, 46)

### 3.4 Accuracy, validity, and hallucination risk

The following accuracy and validity metrics are reported from individual studies and are not pooled estimates. LLM accuracy in sports medicine contexts was heterogeneous across domains and model versions. For athlete sleep management, in a single validation study, content validity ratios ranged from 0.33 for GPT-3.5 to 0.67 for GPT-4 (18). For jet lag management, in the same study, Google Bard achieved a CVR of 0, GPT-3.5 achieved 0.33, and GPT-4 was the only model to reach a statistically acceptable level of validity (CVR = 0.68; *p* < 0.0001 for Bard versus GPT-4) (18). In a focused evaluative study, sample-size calculations demonstrated systematic errors across model versions (17). Orthopaedic rehabilitation guidance accuracy varied substantially by question domain (29). The Reference Hallucination Score instrument classified ChatGPT and Bing as critical hallucination levels, contrasting sharply with Elicit and SciSpace, which were classified as negligible across identical citation-generation tasks (41). Retrieval-augmented generation architectures substantially reduced fabrication rates in clinical NLP tasks (39), and cross-modal attention mechanisms in domain-specific medical AI improved semantic coherence (43). These developments confirm that hallucination is a design-dependent rather than an invariant architectural feature of LLMs, and that engineering investment can progressively reduce it. Their systematic application to sports medicine AI contexts remains an open research priority.

Across included studies, there was evidence that some LLMs perform better than others depending on the task. Newer models such as GPT-4 and GPT-4o tended to perform better than earlier versions (e.g., GPT-3.5) in comparable tasks requiring clinical reasoning, content validity, and structured output generation, although the heterogeneity of the included studies and the absence of meta-analytic comparison preclude a definitive ranking. Retrieval-augmented systems and tools designed for literature search (e.g., Elicit, SciSpace) performed better in reference-intensive tasks due to their ability to reduce hallucinated or fabricated citations. These differences are likely explained by variations in model size, training, data scale, and the integration of an external retrieval mechanism. However, performance remains highly task-dependent, and no single model was consistently superior across all domains. Practically, this suggests that model selection in sports medicine applications should be guided by task type, with general-purpose LLMs used for drafting and explanation tasks, and retrieval-augmented systems preferred for evidence-based or citation-sensitive outputs.

### 3.5 Temporal trends in LLM performance across domains

Across the included studies, there was consistent directional evidence of performance improvement in generative AI and large language models over time, although a formal meta-analytic comparison was not possible due to heterogeneity in evaluation designs and outcome metrics. Newer model versions, particularly GPT-4-based systems, generally demonstrated higher performance than earlier iterations such as GPT-3.5 across comparable sports medicine tasks. For example, GPT-4 achieved higher content validity ratios than GPT-3.5 in sleep and jet lag management guidance for athletes (CVR: 0.67 vs. 0.33 for sleep; 0.68 vs. 0.33 for jet lag) (18).

Similarly, studies using more recent retrieval-augmented generation (RAG) or updated model architectures reported reductions in hallucination frequency and improved factual consistency compared with earlier standalone LLM deployments. However, these trends must be interpreted cautiously, as differences in evaluation frameworks, prompt design, and benchmarking datasets across studies limit direct comparability. Overall, the evidence suggests progressive improvement in model reliability across generations, but with persistent domain-specific limitations in clinical reasoning and sports medicine-specific decision-making tasks.

## 4 Discussion

This scoping review provides the first comprehensive evidence map of GenAI and LLM applications across the full spectrum of sports medicine and athlete health. Thirty-two sources published between January 2023 and March 2026 document both genuine clinical potential and substantive risks in this transition. Three recurring tensions characterise the evidence base: the gap between LLM capability and LLM reliability; the gap between practitioner adoption and the development of governance frameworks; and the gap between the technical sophistication of these tools and the conceptual frameworks available to sports medicine for evaluating them.

### 4.1 LLMs as clinical and coaching support tools: scope and limits

The evidence confirms that LLMs can generate sports medicine outputs broadly aligned with established guidelines when evaluated against general criteria. Resistance training programmes adhered to established periodisation principles (15, 16); exercise prescriptions addressed major safety parameters for chronic conditions (16); nutritional guidance aligned with macronutrient recommendations for common sport categories (26, 27). This pattern is consistent with the broader picture in medicine: LLMs perform adequately at the level of general knowledge representation but struggle with the individualised, context-sensitive, and dynamically adaptive reasoning that expert sports medicine practice requires (9, 11, 48).

The comparison between general LLMs and purpose-built ML architectures is instructive. Wearable biosensor platforms with domain-specific training demonstrated improved recovery-prediction accuracy (4), and the maturation-aware ML framework for adolescent nutritional screening achieved high discriminative accuracy that general LLMs could not match (25). This positions GenAI appropriately within the broader AI-in-sports-medicine landscape: as a broad, accessible, and imprecise tool whose principal value lies in reducing the activation energy for evidence-based practice in under-resourced settings, not as a replacement for validated, domain-optimised clinical decision systems. The narrative review by Zhou and colleagues reinforces this framing, characterising effective AI integration as one that enhances human expertise rather than substituting it (8). Two domains emerge from the available evidence as warranting particular caution for unsupervised LLM deployment: mental health clinical risk assessment and statistical reasoning for research design. In both domains, preliminary evidence derived, respectively, from a simulation study (23) and a focused evaluative study (17) documents confident incorrect outputs that could constitute patient and research safety concerns. These findings require replication under prospective clinical conditions before definitive conclusions can be drawn.

### 4.2 Hallucination, fabrication, and reliability

The hallucination data synthesised in this review constitute among the most clinically urgent findings. Medical AI chatbot evaluation consistently identified reference hallucination and citation fabrication as frequently reported risks in standard ChatGPT deployment (41, 42). A practitioner using ChatGPT to generate a reference list without independent verification faces a material risk of including non-existent citations; a clinician retrieving a treatment guideline through an LLM faces the possibility of receiving confidently stated incorrect information in some evaluations (36, 37). These are structural features of current transformer-based LLMs arising from probabilistic text generation, not marginal reliability concerns that improved prompting resolves.

The AICICA framework (36) extends this concern from episodic error to systematic competency risk. If practitioners habituate to delegating analytical reasoning to LLMs, the professional skills required to detect LLM errors, evaluate the plausibility of AI recommendations, and override inappropriate outputs may erode below the threshold for safe practice. This hypothesis has not been empirically tested among sports medicine practitioners, but its grounding in cognitive offloading research and preliminary evidence on AI-induced phantom expertise (36) is sufficient to justify anticipatory professional development responses. The temporal increase in AI-generated content in published sports medicine research from 2.38 to 6.25% in under 2 years (20) confirms that the research integrity dimension of this problem is active rather than prospective. Editorial AI disclosure policies, author accountability frameworks, and peer review standards require adaptation now. Forensic transparency tools from blockchain-based governance architectures (46) and institutional ethics management systems with real-time verification provide practical implementation pathways.

### 4.3 Ethical implications and governance requirements

The six ethical concern categories identified in this review are interconnected and mutually reinforcing. Algorithmic bias stemming from the underrepresentation of female athletes, youth athletes, and non-Western populations in LLM training data is particularly consequential, as sports medicine practice is increasingly global and population-diverse, while LLM development remains concentrated in North American and East Asian technology ecosystems (12). Bias in training data generates systematic directional errors that favour populations already over-represented in digital health data. The systematic scoping review on AI ethics in sport found no consensus ethical framework addressing these dimensions specifically (12), a gap that professional sports medicine bodies are positioned to close.

Based on limited simulation data, LLMs have produced clinically inappropriate responses across a range of mental health presentations; while these findings warrant caution, they require confirmation in prospective clinical populations.

Data governance concerns are structurally acute in sports medicine because elite athlete biometric data is simultaneously highly sensitive personal health information and commercially valuable performance intelligence. The blockchain-based governance architecture for HIPAA and GDPR-compliant clinical research data exchange (46) provides the technical foundation that institutional frameworks will require. The IOC’s 2019 mental health consensus (31) and the 2024 international consensus on sports psychiatry (32) position mental health screening as requiring human expertise and clinical judgement that current LLMs are not yet able to reliably substitute based on existing evidence. Machine learning analysis of sedentary behaviour (47) and AI applications in exercise and neuroplasticity (35) illustrate that purpose-built tools designed with explicit governance frameworks and validation procedures can extend professional clinical practice rather than compromise it. Governance frameworks that distinguish these responsible deployments from premature use of autonomous LLMs are essential for practitioners seeking to navigate this rapidly evolving landscape.

### 4.4 Future research priorities

Prospective clinical validation studies of LLM accuracy in specific domains of sports medicine decision-making represent the highest methodological priority. The present review, therefore, also provides targeted recommendations for the design of such validation studies across key sports medicine application domains. Simulation-based evaluations capture important information but cannot replicate the practitioner workflow, data quality variation, and time constraints of real clinical settings. Direct testing of the AICICA hypothesis (36) among sports medicine practitioners, measuring whether sustained LLM adoption over 12 to 24 months correlates with detectable reductions in independent diagnostic reasoning as measured by validated competency assessments, would provide the empirical grounding needed for professional standards development.

The recommendations presented in this section are the authors’ proposals for strengthening future evaluation practice, informed by patterns observed across the included evidence; they are not themselves findings of the evidence synthesis and should be read as forward-looking guidance rather than systematic-review conclusions. To improve consistency in how LLMs are evaluated across sports medicine domains, this review also proposes domain-specific minimum evaluation criteria derived from the strongest examples within the included literature, summarised in Table 5. In academic writing and research integrity, structured tools such as the reference Hallucination Score (41) provide a model for quantifying citation accuracy and fabrication risk.

**Table 5:** Recommended minimum evaluation criteria for future LLM validation studies in sports medicine, by application domain.

Domain	Minimum evaluation criteria for future validation studies
Training prescription & exercise programming	Guideline adherence (e.g., alignment with established periodisation principles); safety screening (e.g., contraindications); capacity for individualisation.
Nutritional guidance	Accuracy of macronutrient and hydration recommendations; consistency across repeated outputs; presence of evidence attribution.
Rehabilitation & return-to-sport	Clinical appropriateness; risk-stratification accuracy; integration with objective assessment frameworks.
Mental health	Safe triage behaviour; appropriate referral recommendations; avoidance of harmful responses under simulated clinical scenarios.
Clinical decision support	Diagnostic reasoning accuracy; statistical validity (e.g., correct sample-size calculations); epistemic calibration.
Academic writing & research integrity	Citation accuracy and fabrication risk, quantified using structured tools such as the Reference Hallucination Score (41); mandatory independent verification of all AI-generated references.
Ethics & data governance	Transparency of data provenance and model limitations; bias auditing across athlete subgroups (e.g., sex, age, ethnicity); compliance with data-protection frameworks such as GDPR and HIPAA (46); auditability of AI-assisted decision trails.

More broadly, the field would benefit from a standardised reporting framework for LLM evaluation in sports medicine, analogous to established guidelines such as CONSORT for clinical trials and STARD for diagnostic accuracy studies. Examples from adjacent fields further illustrate the value of structured evaluation: medical licensing examination benchmarks for general medicine AI, BLEU and ROUGE metrics in natural language processing, and retrieval-grounded evaluation protocols for citation accuracy. In addition, governance and accountability frameworks should be operationalised using existing standards, including AI authorship and disclosure guidelines from the Committee on Publication Ethics (COPE), journal-specific policies (e.g., BJSM and JOSPT), and emerging institutional models such as blockchain-based audit systems for research transparency (46). Together, these approaches provide a foundation for more reproducible, comparable, and clinically meaningful evaluation of LLM performance in sports medicine.

Sport-specific LLM benchmarking datasets, analogous to the medical licensing examination datasets that anchor general medicine AI evaluation (49), would enable standardised performance comparisons across model versions and deployment contexts. Multi-domain evaluation platforms that cover training prescription, rehabilitation decision support, nutritional guidance, and mental health triage would substantially strengthen the field’s evidentiary infrastructure. The public health dimension of GenAI in sport, encompassing recreational athletes, youth participants, and community physical activity populations (50, 51), is the least studied despite its population-level significance. Methodological standardisation through a consensus reporting standard for LLM evaluation in sports and exercise medicine, analogous to CONSORT for trials or PRISMA for reviews, would resolve the cross-study heterogeneity that limits this review’s comparative inferences.

### 4.5 Limitations

Several methodological limitations qualify the conclusions of this review. Restricting publications to English introduces a language bias that may exclude relevant evidence from non-English-speaking research communities, including significant AI evaluation activity in East Asian and Arabic-speaking contexts. This restriction may interact with the geographic concentration of the included evidence base, in which Tunisia was the most frequently represented country of corresponding authorship (*n* = 8) and several included sources were co-authored by members of the present review team (Section 2.7); together, the English-language restriction and this authorship network may limit the geographic and linguistic representativeness of the included corpus, and domain-level findings should be interpreted with this potential concentration in mind. Despite systematic searches across six platforms, the rapid growth of the preprint literature in this field may have led to some relevant sources being missed.

In addition, search-string breadth varied across databases because of platform-specific constraints related to Boolean query length, field indexing, and the availability of controlled vocabulary. Consequently, the search strategies used in SPORTDiscus, CINAHL, and Web of Science were necessarily less extensive than those employed in PubMed/MEDLINE and Scopus. Although the searches were developed to maximise relevance and coverage within each platform, this heterogeneity may have reduced retrieval sensitivity and resulted in some potentially eligible studies not being identified.

The heterogeneity of evaluation methodologies across included studies limits the strength of inferences drawn from thematic synthesis; accuracy ranges and hallucination characterisations reflect studies using different benchmarks and should be treated as indicative rather than definitive. As characterised by recent narrative reviews (8), the pace of LLM capability development means that evidence maps such as this one require periodic updating, potentially within 12 to 18 months of publication, to remain actionable for practitioners.

Grey literature, including technical reports, white papers, and institutional policy documents from sports bodies and AI governance organisations, was not searched systematically. This is a meaningful limitation for this field specifically, since practitioner-oriented guidance and institutional decisions on deploying AI in sports medicine are frequently communicated through these applied, non-peer-reviewed channels rather than through indexed journal articles; this omission may contribute to an evidence gap that future reviews should address.

A further limitation relates to the rapid pace of development in generative AI and large language models, which creates a structural “moving-target” problem for evidence synthesis. Studies included in this review, particularly those from earlier in the adoption period (2022–2023), may not fully reflect the capabilities of current model generations. Accordingly, the findings presented here should be interpreted as an evidence baseline rather than a definitive assessment of current state-of-the-art performance. To maintain relevance in such a rapidly evolving field, future research may benefit from adopting living review methodologies with periodic updates. In the interim, practitioners and researchers should prioritise domain-specific validation evidence over general benchmark performance when making deployment decisions, and apply LLM outputs with appropriate expert oversight to mitigate potential risks to patient safety.

## 5 Conclusion

Generative AI and large language models have entered sports medicine at a speed and scale that the available evidence base was not designed to absorb. This scoping review, mapping 32 sources published between January 2023 and March 2026 across seven application domains, documents both potential clinical utility and emerging risks in this transition. LLMs can generate training programmes, dietary guidance, rehabilitation content, and clinical information aligned with established guidelines at a level useful for expert-supervised augmentation; they cannot yet do so with the accuracy, reliability, or contextual precision required for autonomous clinical deployment. Medical AI chatbot hallucination has been classified as critical in some evaluations of ChatGPT and Bing, and together with the absence of epistemic calibration in LLM outputs may represent a patient safety concern in unsupervised deployment scenarios.

Three conclusions carry immediate practical weight:

1. LLMs should be positioned as supervised decision-support assistants requiring expert review and individualisation, not as autonomous clinical or coaching advisors; the current evidence does not support a higher level of delegation.
2. Governance frameworks addressing data privacy, algorithmic bias, professional accountability, and research integrity must be developed and operationalised before these tools are embedded in athlete health pathways; the technical architectures to support such frameworks exist but have not yet been deployed at scale in sports medicine contexts.
3. The sports medicine community requires standardised benchmarking datasets, prospective clinical validation studies, and a consensus reporting standard for LLM evaluation to build the evidentiary infrastructure needed for evidence-based deployment decisions.

The opportunities that GenAI offers to reduce barriers to evidence-based practice in under-resourced settings, extend professional reach across recreational and community populations, and accelerate the translation of research into practice are real. Realising them requires the same commitment to validation, transparency, and patient safety that governs every other technological advance in sports medicine.

However, several of these findings are derived from limited or simulation-based studies and require confirmation in prospective real-world clinical settings.
